# Where in 1901?

**Published:** 1900-07

**Authors:** 


### WHERE IN 1901?

THE East has had many meetings of the A. V. M. A. The Central West, the Northwest, the region beyond the Mississippi, and the South have all felt the valued impulse of Association conventions in their midst, and there remains but one section of our territory that needs and yearns for our presence, and which should have our most serious consideration. We refer to the Pacific coast. And why not cross the continent in 1901? With this attained we could surely claim beyond any question the name of A. V. M. A., and in strengthening our colleagues' hands in that great and wonderful section of our country we would likewise strengthen our own. Will the Pacific slope veterinarians lead in the movement?

